# Effect of esketamine on postoperative analgesia and postoperative delirium in elderly patients undergoing gastrointestinal surgery

**DOI:** 10.1186/s12871-024-02424-w

**Published:** 2024-02-01

**Authors:** Jing Liu, TingTing Wang, Jian Song, Li Cao

**Affiliations:** Department of Anesthesiology, The Affiliated Taian City Central Hospital of Qingdao University, No. 29 Longtan Road, Taishan District, Tai’an City, Shandong Province 271000 China

**Keywords:** Anti-inflammatory, Esketamine, Postoperative analgesia, Postoperative delirium

## Abstract

**Objective:**

To investigate the analgesic effect of esketamine combined with low-dose sufentanil in elderly patients after gastrointestinal surgery, and whether the anti-inflammatory effect of esketamine is involved in the mechanism of postoperative delirium.

**Method:**

We enrolled sixty elderly patients (age ≥ 65 years old, American Society of Anesthesiologists (ASA) grade I-III) who underwent gastrointestinal surgery. Patients were randomly assigned to Group C (control group) who received sufentanil 2 ug/kg, and Group E (experimental group) who received sufentanil 1.5 ug/kg + esketamine 1 mg/kg, with 30 patients in each group. All patients underwent total intravenous anesthesia during the surgery and were connected to a patient-controlled intravenous analgesia (PCIA) pump after surgery. The primary outcome was the evaluation of pain at 4, 24, 48 h after surgery which was evaluated by NRS scores. In secondary outcomes, inflammation was assessed by measuring IL-6 levels using ELISA. The postoperative delirium and the occurrence of adverse reactions were observed on the 1st and 3rd day after surgery.

**Results:**

The NRS scores at 4, 24, and 48 h after surgery in the experimental group [(4.53 ± 1.22), (3.46 ± 0.73), (1.37 ± 0.99)] were lower than that in the control group [(5.23 ± 1.16), (4.46 ± 0.77), (2.13 ± 0.78)] (*P* < 0.05). The concentration of serum IL-6 in the experimental group at 24 and 48 h after operation [(15.96 ± 4.65), (11.8 ± 3.24)] were lower than that in the control group [(23.07 ± 4.86), (15.41 ± 4.01)] (*P* < 0.05); the incidence of postoperative delirium in the experimental group was less than that in the control group (*P* < 0.05); there was no significant difference in the incidence of postoperative nausea and vomiting between the two groups (*P >* 0.05), and neither group had nightmares or delirium.

**Conclusion:**

Esketamine may enhance postoperative pain management compare with sufentanil, and esketamine has anti-inflammatory effects that reduce the incidence of postoperative delirium.

**Trial registration:**

Full name of the registry: Chinese Clinical Trial Registry. Trial registration number: ChiCTR2300072374. Date of registration:2023/06/12

**Supplementary Information:**

The online version contains supplementary material available at 10.1186/s12871-024-02424-w.

## Introduction

With advances in medical technology, an increasing number of elderly patients are opting for surgical treatments to address ailments. As a result, there is growing concern among patients and healthcare professionals about anesthesia-related post-operative complications, such as postoperative delirium (POD), which is mainly an acute neurological disorder occurring within 2 to 5 days after surgery. It is manifested in reduced clarity of conscious content, accompanied by disturbance of awakening-sleep cycle and psychomotographic behavior disorders. Postoperative delirium is postoperative, and patients have no contact with the surrounding environment and impaired ability to understand themselves. Thinking, memory, understanding and judgment are impaired, speech is incoherent and disordered, orientation is impaired, gibberish is gibberish, excitement is agitated. In addition, there are obvious hallucinations and delusions [1]. Studies have indicated that there is a two to three-fold increased risk of POD complications and a two to three-fold increased risk of perioperative mortality, as well as prolonged hospitalization and increased medical expenses during hospitalization [2–4]. A long-term follow-up study revealed increased incidence of long-term postoperative cognitive dysfunction, decreased quality of life, and increased long-term mortality among patients with POD [5]. In this study, we used the postoperative analgesic effect of esketamine combined with sufentanil in patients to evaluate the analgesic effect and observe the impact of esketamine on the inflammatory response by measuring IL-6 levels; we also explored whether the anti-inflammatory effect of esketamine is involved in the mechanism of POD and its influence on POD. This study aims to improve the analgesic effect for elderly patients after gastrointestinal surgery, as well as their postoperative cognitive function, thereby improving their postoperative quality of life and reducing the burden on families and society.

## Data and methods

### General data

This was a single-center prospective, randomized, controlled, and double-blind clinical trial. After obtaining approval from the Ethics Committee of Tai’an Central Hospital(2021-06-62), 60 patients (age ≥ 65 years old, ASA grade I-III) who had undergone laparoscopic gastrointestinal surgery (gastric cancer, colon cancer, and rectal cancer) at Tai’an Central Hospital from November 2021 to November 2022 were enrolled in this study. The types of surgery has been showed in the Table 1.

**Table 1:** The types of surgery

Group	Group C	Group E
**Gender (Male/Female)**	18/12	21/9
Laparoscopic radical gastrectomy	16	13
Laparoscopic radical resection of colon cancer	8	10
Laparoscopic radical resection of rectal cancer	6	7

The exclusion criteria were as follows: Patients with severe internal diseases, endocrine and immune system diseases, neurological and mental diseases (Patients with cerebrovascular disease, cerebral infarction, cerebral hemorrhage, currently suffering from Parkinson’s disease, craniocerebral occupying patients. We also excluded patients with diagnosed mental illness, such as depression, mania, neurasthenia and sleep disorders), long-term drug abuse, or intolerance or allergy to experimental drugs. Patients with intraoperative blood transfusion were also excluded. A simple cognitive function test (3-minute Diagnostic Interview for Confusion Assessment Method (CAM)) (Supplementary material) was performed for each of our patients at the pre-anaesthesia visit. All patients voluntarily signed the informed consent form before the surgery and received postoperative patient-controlled intravenous analgesia (PCIA).

### Methods

1) Grouping: Patients were randomized to either group C (control group, sufentanil group) or group E (sufentanil + esketamine group) based on the SPSS-generated table of random numbers, which was stored in sealed envelopes. A resuscitation room nurse, who was not involved in data collection, configured postoperative analgesic pumps based on random numbers. The doctor who performed anesthesia and surgery, another resuscitation room nurse who was responsible for data collection, and the patient were blinded to the group assignments.

2) Anesthesia: All patients fasted for 12 h and avoided all liquids for 6 h before surgery. After entering the operating room, the upper limb vein passage was established, and the patients were connected to monitors to track their vital signs such as electrocardiogram (ECG), non-invasive blood pressure (NIBP), peripheral capillary oxygen saturation (SPO2), heart rate (HR), respiratory rate (RR), bispectral index monitoring (BIS). In all patients, intraoperative blood pressure fluctuated within the range of ± 20% of the basic blood pressure. In case of hypotension, deoxyadrenaline or ephedrine was used to boost blood pressure.

Anesthesia induction: Intravenous administration of midazolam 0.05 mg/kg, etomidate 0.3 mg/kg, sufentanil 0.4 µg/kg, and rocuronium bromide 0.8 mg/kg was performed. Continuous mechanical ventilation was used to control respiration; the settings were 8 ml/kg, 12 times/min, 1:2 standard settings for tidal volume, respiratory rate, and inspiratory to expiratory ratio.

Anesthesia maintenance: Propofol and remifentanil were continuously pumped at 2–5 mg/(kg h) and 0.1–1 µg/ (kg min), respectively. Propofol was discontinued 10 min before the end of the surgery, while remifentanil was discontinued 5 min before the end of the surgery. Each patient was monitored using BIS, which was maintained between 40 and 65 during anesthesia. Neostigmine (0.2 mg/kg) and atropine (0.1 mg/kg) were used to antagonize muscle relaxants after surgery,.Nerve blocks and local regional anesthesia were not used during the procedure, nor were analgesics such as morphine used, and no antiemetic drugs were given during perioperative period.

3) Analgesia. Patients were connected to the PCIA pump by specialized nurses after entering the recovery room at the end of anesthesia, according to group assignments.

Group C: Sufentanil 2 ug/kg analgesic formula.

Group E: 1.5 ug/kg sufentanil + esketamine 1 mg/kg analgesic formula; both groups of drugs were diluted to 100 ml with normal saline.

PCIA parameter setting: When the patient pressed the PCIA pump button, a 2 ml load of analgesic was administered at a background infusion rate of 2 ml/h and a pump infusion volume of 2 ml/time; the PCIA had a locking time of 15 min. PCIA pumps were used until 48 h after the surgery.

### Observation indicators

#### Primary indicators

Postoperative pain was evaluated by Numerical rating scale scores (NRS scores) at 4, 24, and 48 h after surgery (0 is painless, 1–3 is mild pain, does not affect sleep; 4–6 is moderate pain; 7–9 are classified as severe pain, difficulty falling asleep or pain waking up during sleep; 10 is excruciating pain, unbearable).

#### Secondary indicators

3 ml peripheral venous blood was drawn before induction, after surgery, 24 h after surgery and 48 h after surgery. Interleukin (IL-6) concentration of peripheral venous blood was detected by radioimmunoassay (after the blood samples were centrifuged, the extracted serum was stored at -18℃ and then uniformly tested by ELESA); the incidence of postoperative delirium on the 1st and 3rd day after surgery was assessed by CAM, which was shown in Supplementary material; the incidence of postoperative nausea and vomiting was also observed in the study.

**Fig. 1:**
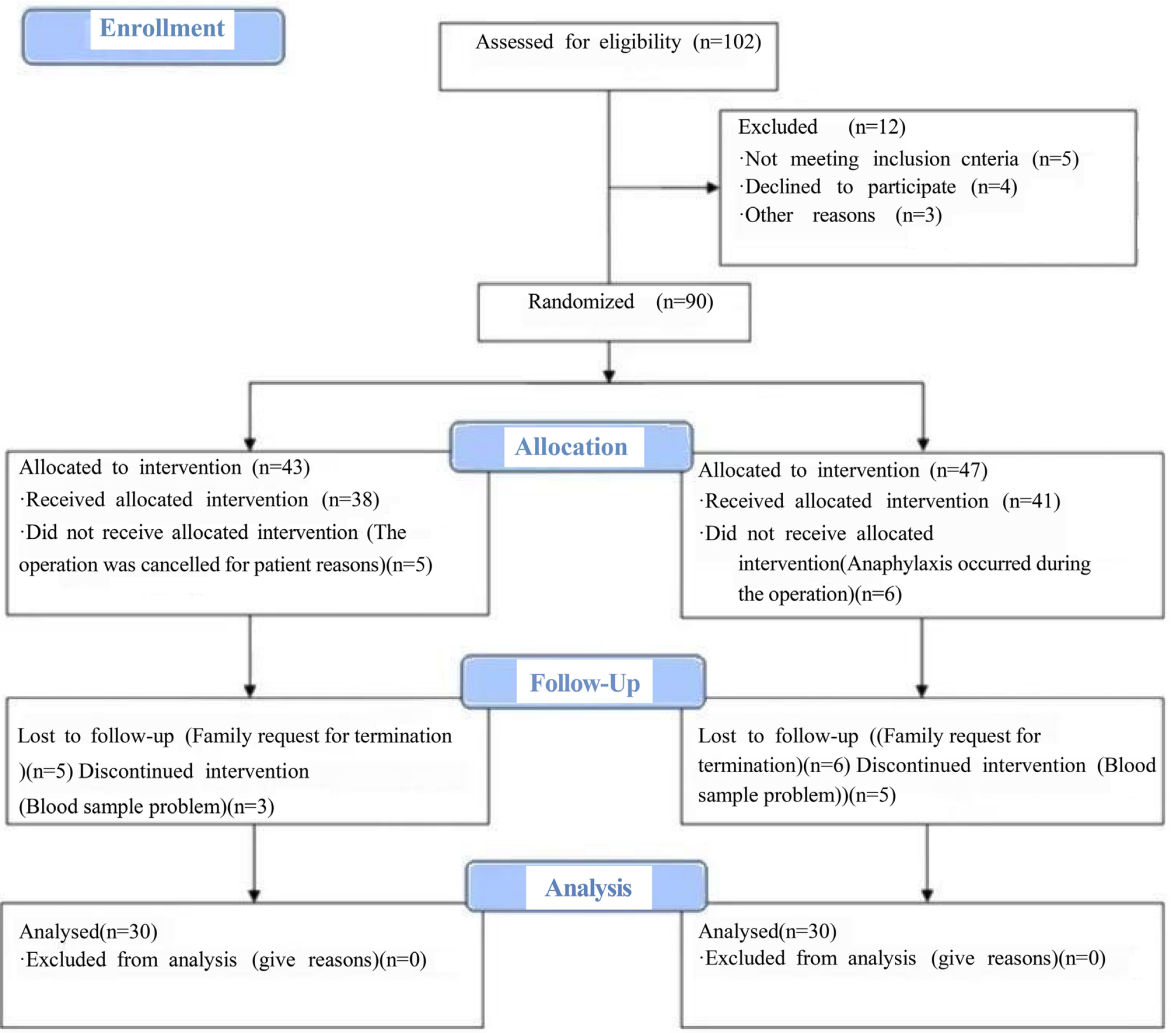
The CONSORT flow diagram of the whole research process

The flow chart of the whole research process was shown in Fig. 1.

### Statistical methods

Sample size calculation: According to the preliminary experiment, the 12 h postoperative postoperative analgesia for gastric cancer patients using sufentanil or sufentanil combined with esketamine, the postoperative 12hNRS scores were about 3.2 ± 1.5 and 2.6 ± 1.4, respectively, with 80% efficacy and double-tail α = 0.05, considering the 20% exit rate. Calculated by G-power3.1 software, 60 participants are required. The data were statistically analyzed using SPSS25.0 software. The measurement data is expressed as mean ± standard deviation or median (P25, P75), and the comparison between groups was performed by either the two independent samples t-test or the Wilcoxon Mann-Whitney two-sample rank-sum test. The paired t-test was used for within-group comparisons. Enumeration data were analyzed using the chi-squared test or Fisher’s exact probability method, and the difference is statistically significant when *P* < 0.05.

## Results

### Primary indicators

#### NRS scores at 4, 24, and 48 h after surgery

Compared with the control group [(5.23 ± 1.16), (4.46 ± 0.77), (2.13 ± 0.78)], the NRS scores at 4, 24, and 48 h after surgery in the experimental group were significantly lower [(4.53 ± 1.22), (3.46 ± 0.73), (1.37 ± 0.99)] (*P* < 0.05), especially at 24 h (*P* < 0.001) and 48 h (*P* < 0.01) after surgery, the difference between the two groups was more significant. It means that, at 4 h, 24 and 48 h, the postoperative analgesia effect of experimental group was significantly better than that of control group. (Table 2).

**Table 2:** Comparison of numerical rating scale (NRS) scores of postoperative analgesia between the two groups (x ± s)

Time	Control group	Experimental group	Difference and 95% confidence interval (CI)	T-test	
				***T*** value	***P*** value
4 h after surgery	5.23 ± 1.16	4.53 ± 1.22	0.7 (0.08–1.32)	2.26	0.027
24 h after surgery	4.46 ± 0.77	3.46 ± 0.73	1.0 (0.61–1.39)	5.14	< 0.001
48 h after surgery	2.13 ± 0.78	1.37 ± 0.99	0.77 (0.31–1.23)	3.31	0.002

### Secondary indicators

#### Concentration of interleukin-6 (IL-6) in peripheral venous blood before induction, immediately after operation, and at 24 and 48 h after surgery

The concentration of IL-6 in the serum of the two groups increased after the operation, reached the highest value at 24 h after operation, and began to decrease at 48 h after operation.

There was no statistical significance in the concentration of interleukin-6 (IL-6) in serum between the two groups before induction and immediately after surgery.

At 24 and 48 h after surgery, the concentration of peripheral venous interleukin-6 (IL-6) in the experimental group [(15.96 ± 4.65), (11.8 ± 3.24)] was statistically significantly lower than that in the control group [(23.07 ± 4.86), (15.41 ± 4.01)] (*P* < 0.01) (Table 3).

**Table 3:** The ratio of serum IL-6 concentrations between the two groups at different time points (x ± s)

Time	Control group (pg/ml)	Experimental group (pg/ml)	Difference and 95% confidence interval (CI)	T-test	
				***T*** value	***P*** value
Preoperative	2.43 ± 0.56	2.28 ± 0.59	0.15 (0.38–0.67)	0.58	0.569
Immediately after operation	8.97 ± 1.22	8.96 ± 1.02	0.01 (1.04–2.07)	0.03	0.977
24 h after surgery	23.07 ± 4.86	15.96 ± 4.65	7.1 (2.89–11.3)	3.54	0.002
48 h after surgery	15.41 ± 4.01	11.8 ± 3.24	3.61 (1.4-7.0)	2.18	0.042

#### Incidence of postoperative delirium on the 1st day and 3rd day after the surgery

The incidence of postoperative delirium was statistically significantly lower on the 1st and 3rd day after surgery in the experimental group [12(40) /4(13.3)] (*P* < 0.05) (Table 4).

**Table 4:** Comparison of the incidence of postoperative delirium between the two groups

Grouping	Total (Case)	Delirium [Case (%)]	No delirium [Case (%)]	Difference and 95% CI	Chi-squared test	
					Chi-squared value	***P*** value
Control group	30	12(40)	18(60)	26.7 (20.5–46.3)	4.176	0.041
Experimental group	30	4(13.3)	26(86.7)			

#### Incidence of postoperative nausea and vomiting

The number of patients with postoperative nausea and vomiting was high in both groups. There was no significant difference in the incidence of postoperative nausea and vomiting between the experimental group and the control group [22(73.3)/18(60)] (*P* > 0.05) (Table 5).

**Table 5:** Comparison of the incidence of postoperative nausea and vomiting between the two groups

Grouping	Total (Case)	Nausea and vomiting [case (%)]	No nausea and vomiting [case (%)]	Chi-squared test	
				Chi-squared value	***P*** value
Control group	30	22(73.3)	8(26.7)	1.200	0.273
Experimental group	30	18(60)	12(40)		

## Discussion

The result of our study shower that compared with group C, the analgesic effect of group E had obvious statistical difference, and the analgesic effect was exact. What’s more, in group E, the dosage of sufentanil was lower than that of the control group. In practical clinical work, multimodal analgesia is the first choice for perioperative analgesia, and PCIA is also a part of multimodal analgesia. This study confirmed the analgesic effect of esketamine. Although the difference in the analgesic score between the two groups was less than 1, our purpose was not only to prove the analgesic effect of esketamine, but also whether it could reduce the use of opioids, so as to reduce the effects of opioids on postoperative cognition and adverse reactions such as nausea and vomiting in elderly patients. Esketamine can reduce the use of opioids, and also provide a better choice for postoperative PCIA administration and matching in elderly patients. In this study, we combined esketamine with sufentanil to observe the postoperative analgesic effect and serum IL-6 concentration, as well as the reduction of pain and inflammation levels, thus reducing the incidence of postoperative delirium in elderly patients. The combination of esketamine with low-dose sufentanil not only achieves a more effective analgesic effect, but it also reduces the dosage of opioids. In this study, we also explored differences in postoperative adverse reactions such as nausea and vomiting between the two groups. The results for nausea and vomiting show that there is no statistical difference between the two groups.

Given the evidence-based and consensus based risk factors for delirium put forward by the European Society of Anesthesiology, the pathogenesis of postoperative delirium includes: Preoperative, intraoperative, and postoperative factors [6–8]. The patients’ condition is related to preoperative factors and is not easily affected by external factors. Intra-operative factors and operative factors are related to anesthesia administration and can be reduced or avoided by the use of certain intervention measures; postoperative factors are related to the treatment of patients in the postoperative rehabilitation stage and are important risk factors for delirium. Therefore, intervention in patients at the postoperative stage was chosen in this study.

Pain is the most common postoperative complication. Observational studies found that a higher postoperative pain score was associated with an increased risk of delirium [9–11]. It means that the greater the pain severity, the greater the physical trauma, and higher the risk of delirium [12]. In addition, the use of opioids, especially long-acting opioids, is also related to the increased risk of POD [13]. As we all know, POD is caused by the interaction of multiple risk factors (such as pain, opioids, sleep deprivation, and inflammation), which presents challenges for its prevention and treatment [14]. However, the pathophysiological mechanism of delirium is not well understood, and neuroinflammation remains the dominant research topic. Systemic inflammatory mediators increased significantly following surgery and remained elevated throughout the postoperative period. Studies have shown that serum IL-6 and CRP levels in patients with POD are higher than those in patients without POD [15]. During the early postoperative period, an increase in peripheral blood IL-6 concentration is associated with a higher risk of postoperative delirium [16–18]. As a result, it can be deduced that early serum inflammatory variables may be POD predictors.

Clinical studies have shown that peripheral inflammation can cause a loss of structural and functional integrity of the blood-brain barrier [19], with inflammatory cells and mediators then transferring to the central nervous system [20]. Accumulation of inflammatory mediators leads to loss of synaptic plasticity [21], apoptosis of nerve cells [22], and impaired neurogenesis [23]. Eventually, it leads to postoperative delirium. The results of this study also revealed that the concentration of IL-6 in the peripheral blood of patients started to rise and peaked at 24 h after the surgery and showed a downward trend at 48 h after the surgery. Compared to the experimental group, the control group had a higher IL-6 concentration and a higher incidence of delirium.

Studies have also shown that effective pharmacological methods can reduce the incidence of delirium [24]. According to the pathophysiological overlap between inflammation pain and neuronal damage, anti-inflammatory analgesics are the best choice to prevent delirium in patients with acute postoperative pain. Ketamine, an intravenous anesthetic, has been used clinically since 1970. It has good analgesic, sedative, anti-inflammatory, and antidepressant effects [25]. In recent years, ketamine has been used as a part of multimodal analgesia to treat acute pain, and its fewer side effects have attracted attention [26]. Ketamine has a multi-pathway mechanism and inhibits the release of inflammatory factors such as bradykinin, significantly inhibits TNF-α activity, reduces the production of inflammatory cytokines such as IL-6, and inhibits the inflammatory response [27]. Esketamine, one of the ketamine isomers, is twice as potent as ketamine [28]. Both ketamine and esketamine have been shown to reduce inflammation in the nervous system by inhibiting the inflammatory factor pathway [29]. 

Several studies have indicated that esketamine reduces acute postoperative pain [30–32]. However, it remains unclear whether the use of esketamine reduces postoperative opioid consumption. In addition, there were some limitations in the study. Frst, there were some shortcomings in the design, for example, two more groups can be added in the study, including one group which given esketamine 1.0 mg/kg alone and the other that given esketamine 1.5 mg/kg or 2.0 mg/kg. If the analgesic effect is better than sufentanil 1.5ug/kg + esketamine 1.0 mg/kg, and there are no side effects of esketamine such as hallucinations, nightmares, etc., and even the adverse reactions of opioid nausea and vomiting are reduced, it can be more clear that esketamine has a good analgesic effect and reduces the dosage of opioids. Second, there was no significant difference in postoperative nausea and vomiting between the two groups, which may be caused by the small sample size in our analysis. And last, the number of times the patient pressed the analgesic pump after surgery was not recorded by the computer, resulting in the loss of a good comparative indicator.

In future studies, we will expand the sample size and the sufentanil dosage in the experimental group could be reduced prior to comparison.

## Conclusion

These results indicate that compared with sufentanil, esketamine for postoperative analgesia in elderly patients may has a good analgesic effect. Esketamine can alleviate the inflammatory response and reduce the incidence of POD, thereby improving the postoperative quality of life for patients.

### Electronic supplementary material

Below is the link to the electronic supplementary material.

Supplementary Material 1

## Data Availability

All data generated or analysed during this study are included in this article. Further enquiries can be directed to the corresponding author.
