# Supplementary material for: Effect of esketamine on postoperative analgesia and postoperative delirium in elderly patients undergoing gastrointestinal surgery
Source: BMC Anesthesiol. 2024 Feb 1;24:46. doi: 10.1186/s12871-024-02424-w (PMC10832082; doi:10.1186/s12871-024-02424-w)
Supplement: Supplementary file 1 — Supplementary Material 1 [file 12871_2024_2424_MOESM1_ESM.doc]

# Confusion Assessment Method (CAM)

| **The diagnosis of delirium by CAM requires the presence of BOTH feature A and B** | | |
| --- | --- | --- |
| **CAM**  **Confusion Assessment Method** | **A.**  **Acute onset**  and  **Fluctuating course** | Is there evidence of an acute change in mental status from patient baseline?  Does the abnormal behaviour:   - come and go? - fluctuate during the day? - increase/decrease in severity? |
|  | **B.**  **Inattention** | Does the patient:   - have difficulty focusing attention - become easily distracted? - have difficulty keeping track of what is said? |
|  | **AND the presence of EITHER feature C or D** | |
|  | **C.**  **Disorganized thinking** | Is the patient’s thinking   - disorganized - incoherent   For example does the patient have   - rambling speech/irrelevant conversation? - unpredictable switching of subjects? - unclear or illogical flow of ideas? |
|  | **D.**  **Altered level of consciousness** | Overall, what is patient’s level of consciousness:   - alert (normal) - vigilant (hyper-alert) - lethargic (drowsy but easily roused) - stuporous (difficult to rouse) - comatose (unrousable) |
